# Correction: Nguyen et al. Prognostic Significance of Key Molecular Markers in Thyroid Cancer: A Systematic Literature Review and Meta-Analysis. *Cancers* 2025, *17*, 939

**DOI:** 10.3390/cancers18081255

**Published:** 2026-04-16

**Authors:** Linh T. T. Nguyen, Emma K. Thompson, Nazim Bhimani, Minh C. Duong, Huy G. Nguyen, Martyn Bullock, Matti L. Gild, Anthony Glover

**Affiliations:** 1 Kolling Institute, Northern Sydney Local Health District and Sydney Medical School, Faculty of Medicine and Health, University of Sydney, Sydney, NSW 2050, Australia; thng0079@uni.sydney.edu.au (L.T.T.N.); martyn.bullock@sydney.edu.au (M.B.); matti.gild@sydney.edu.au (M.L.G.); 2 Department of Endocrinology, The 108 Military Central Hospital, Hanoi 100000, Vietnam; 3 The Kinghorn Cancer Centre, Garvan Institute of Medical Research, St. Vincent’s Clinical School, Faculty of Medicine, University of New South Wales, Darlinghurst, NSW 2010, Australia; e.thompson.1@unsw.edu.au (E.K.T.); nazim.bhimani@sydney.edu.au (N.B.); 4 Specialty of Surgery, Sydney Medical School, Faculty of Medicine and Health, The University of Sydney, Camperdown, NSW 2050, Australia; 5 School of Population Health, University of New South Wales, Sydney, NSW 2033, Australia; minh.duong@unsw.edu.au; 6 School of Biomedical Engineering, University of Technology Sydney, Sydney, NSW 2007, Australia; gia.h.nguyen@student.uts.edu.au; 7 Department of Endocrinology, Royal North Shore Hospital, Northern Sydney Local Health District, Sydney, NSW 2065, Australia

**Error in Figure**

In the original publication [1], there was a mistake in Figure 1, “Selection and screening process for included studies from three databases”, as published.

- At the Identification step, 20 documents from sources other than the three databases were all manually excluded. This step is added to one branch of the flowchart to accurately reflect the process.
- At the Eligibility assessment step, the number of studies excluded was 123, not 107. This correction does not affect the final number of studies included, which remains 9.

The corrected Figure 1 appears below. The authors state that the scientific conclusions are unaffected. This correction was approved by the Academic Editor. The original publication has also been updated.

## Figures and Tables

**Figure 1:**
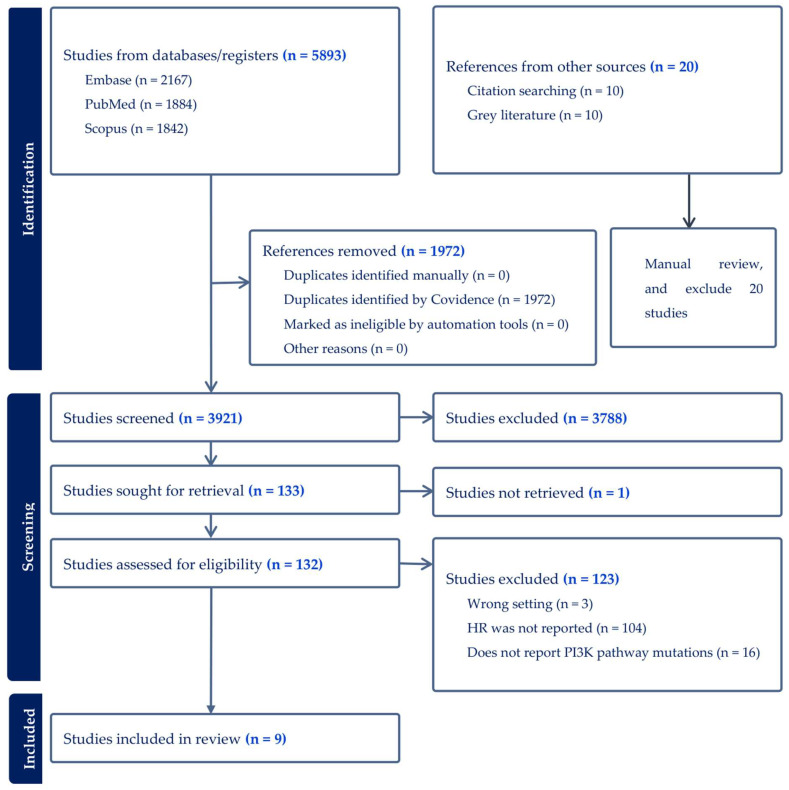
Selection and screening process for included studies from three databases.
