# Supplementary material for: Prognostic Significance of Key Molecular Markers in Thyroid Cancer: A Systematic Literature Review and Meta-Analysis
Source: Cancers (Basel). 2025 Mar 10;17(6):939. doi: 10.3390/cancers17060939 (PMC11940365; doi:10.3390/cancers17060939)
Supplement: Supplementary file 1 [file cancers-17-00939-s001.zip › Supplement S1_Search summary.pdf]

SEARCH STRATEGY CONCEPT

**Concept**	**Thyroid cancer**	**Mutations**	**Survival**
**Keywords**	Thyroid AND<br>cancer*, neoplasm*, tumor, tumors, malignan*, carcino*, metast*<br><br>Narrow: "Thyroid cancer*"<br><br>…And other related terms	Mutation*<br><br>Particular mutations?<br>TERT<br>PI3K<br>TP53<br><br>…And other related terms	Prognos*<br>(Finds prognosis, prognostic etc.)<br><br>Survival<br><br>…And other related terms
**Subject headings**	"Thyroid Neoplasms" [Mesh]<br><br>"Thyroid Neoplasms/genetics" [Mesh]	"Mutation" [Mesh]<br><br>Mutation/genetics [Mesh]	Prognosis [Mesh]<br><br>"Survival analysis" [Mesh}
All searches were restricted to the last 10 years i.e. 2013 – 2023			

SEARCH STRATEGIES FOR EACH DATABASE

1. PubMed:

- **Keywords:** The search included the following keywords combined with Boolean operators (AND, OR) to maximize retrieval:

  Thyroid AND (cancer OR neoplasm* OR tumo* OR malignan* OR carcino* OR metast*) AND (Mutation* OR PI3K* OR PTEN OR AKT* OR mTOR* OR PIK3* OR TERT) AND (Prognos* OR survival OR Recurren* OR Progression)*

- **MeSH Terms:** Specific MeSH (Medical Subject Headings) terms were also used:

  "Thyroid Neoplasms"[MeSH], "Thyroid Neoplasms/genetics"[MeSH], "Mutation"[MeSH], "Mutation/genetics"[MeSH], "Prognosis"[MeSH], "Survival analysis"[MeSH]

2. Embase:

- **Keywords:** The search was conducted using keywords with Boolean operators:

  Thyroid AND (cancer* OR neoplasm* OR tumo* OR malignan* OR carcino* OR metast*) AND (Mutation* OR PI3K* OR PTEN OR AKT* OR mTOR* OR PIK3* OR TERT) AND (Prognos* OR survival OR Recurren* OR Progression)

- **Emtree Terms:** Relevant Emtree terms were incorporated to improve the precision of the search:

  'Thyroid Neoplasms'/exp, 'Thyroid Neoplasms/genetics'/exp, 'Mutation'/exp, 'Mutation/genetics'/exp, 'Prognosis'/exp, 'Survival analysis'/exp

3. Scopus:

- **Keywords:** The search used the following keyword combinations:

  (TITLE-ABS-KEY("Thyroid" AND (cancer OR neoplasm* OR tumo* OR malignan* OR carcino* OR metast*)) AND TITLE-ABS-KEY((Mutation* OR PI3K* OR PTEN OR AKT* OR mTOR* OR PIK3* OR TERT)) AND TITLE-ABS-KEY((Prognos* OR survival OR Recurren* OR Progression)))*
- **Subject Areas:** Filters were applied to focus on relevant subject areas.

SEARCH RESULTS SCREENSHOT OF EACH DATABASE

PubMed N = 1,884

Search	Actions	Details	Query	Results	Time
#12	•••	>	Search: **#3 AND #7 AND #10** Filters: **from 2013 - 2023**	1,884	18:07:42
#11	•••	>	Search: **#3 AND #7 AND #10**	2,529	18:07:08
#10	•••	>	Search: **#8 OR #9**	1,838,536	18:05:12
#9	•••	>	Search: **(prognos*[Title/Abstract]) OR (survival[Title/Abstract])**	1,736,616	18:04:20
#8	•••	>	Search: **"Survival Analysis"[Mesh]**	334,566	18:02:34
#7	•••	>	Search: **#4 OR #5 OR #6**	1,157,222	18:00:38
#6	•••	>	Search: **TERT*[Title/Abstract] OR PI3K*[Title/Abstract] OR TP53*[Title/Abstract]**	362,475	17:58:59
#5	•••	>	Search: **mutation*[Title/Abstract]**	769,445	17:58:32
#4	•••	>	Search: **"Mutation/genetics"[Mesh]**	116,341	17:51:29
#3	•••	>	Search: **#1 OR #2**	83,835	17:50:23
#2	•••	>	Search: **(thyroid[Title/Abstract]) AND ((cancer*[Title/Abstract] OR neoplasm*[Title/Abstract] OR tumor*[Title/Abstract] OR malignan*[Title/Abstract] OR carcino*[Title/Abstract] OR metast*[Title/Abstract]))**	83,247	17:48:13
#1	•••	>	Search: **"Thyroid Neoplasms/genetics"[Mesh]**	9,475	17:45:28

Embase N = 1,364

# ▲	Searches	Results	Type
1	thyroid cancer.mp. or *thyroid cancer/	60701	Advanced
2	*mutation/ or mutation.mp.	1195923	Advanced
3	TERT.mp.	73425	Advanced
4	*"Pi3K/Akt signaling"/ or PI3K.mp.	96748	Advanced
5	TP53.mp.	45532	Advanced
6	2 or 3 or 4 or 5	1358843	Advanced
7	*cancer prognosis/ or prognosis.mp.	1276597	Advanced
8	*cancer survival/ or *disease free survival/ or *cancer specific survival/ or *overall survival/ or *mean survival time/ or *median survival time/ or *disease specific survival/ or *cancer free survival/ or *survival analysis/ or survival.mp.	2233574	Advanced
9	7 or 8	3029362	Advanced
10	1 and 6 and 9	2645	Advanced
11	limit 10 to yr="2013 -Current"	2167	Advanced

Scopus N = 1,959

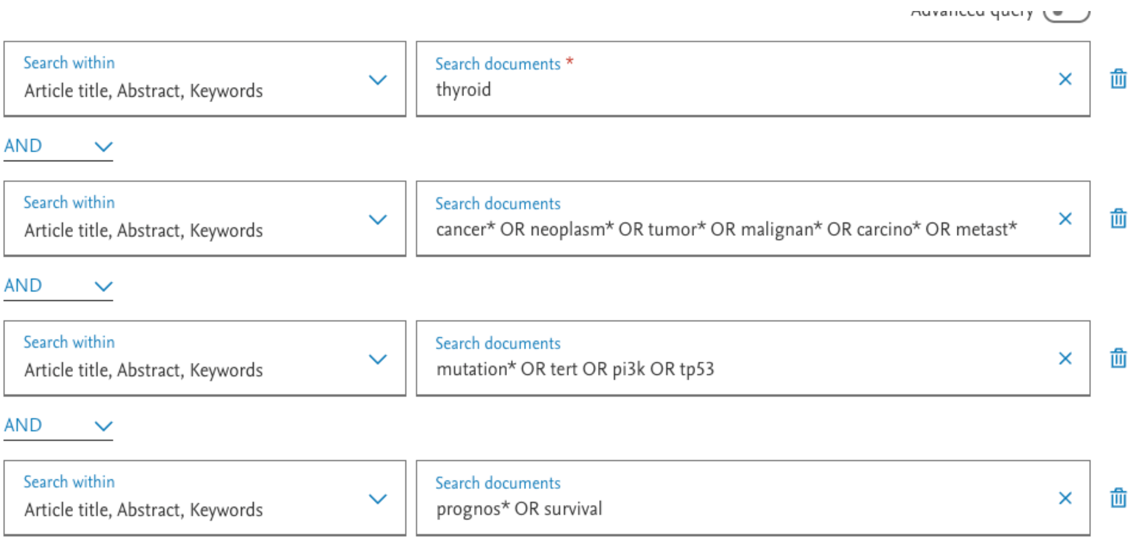

SCREENING

1. Inclusion criteria

(1) Follicular derived thyroid carcinomas, including well-differentiated and poorly differentiated subtypes
(2) Documented survival outcomes including OS
(3) Patients underwent NGS molecular testing using gene panels including *BRAF*, *RAS*, *TERT*, *PI3K/AKT* or *TP53* mutations
(4) Studies had follow-up of at least 6months for ATC and 1 year for DTC
(5) Full-text studies
(6) Peer-review studies

2. Exclusion criteria

(1) The focus was only on MTC
(2) The study did not report on OS and only reported clinicopathological outcomes
(3) The study had inadequate follow-up time
(4) Tumours were only sequenced for BRAF mutations without secondary co-mutations

3. Process

- Search results were imported to Covidence
- Duplicates were removed using Covidence.
- Titles and abstracts were screened for relevance.
- Full-text articles were retrieved and assessed for eligibility.
