# Supplementary material for: Prognostic Significance of Key Molecular Markers in Thyroid Cancer: A Systematic Literature Review and Meta-Analysis
Source: Cancers (Basel). 2025 Mar 10;17(6):939. doi: 10.3390/cancers17060939 (PMC11940365; doi:10.3390/cancers17060939)

SENSITIVITY ANALYSIS

Figure S1: Sensitivity analysis for BRAF on OS

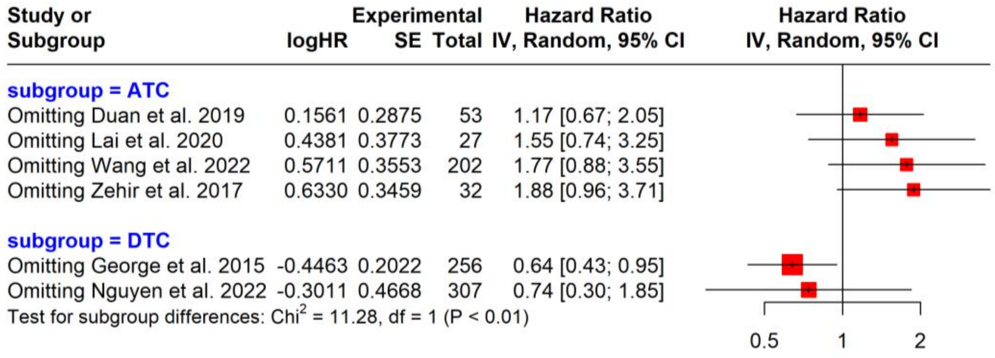

Figure S2: Sensitivity analysis for TERT on OS

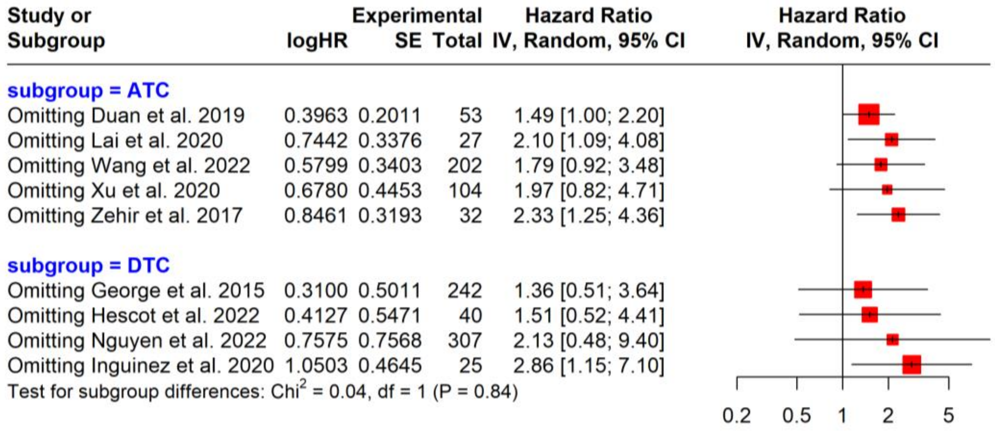

Figure S3: Sensitivity analysis for PI3K on OS

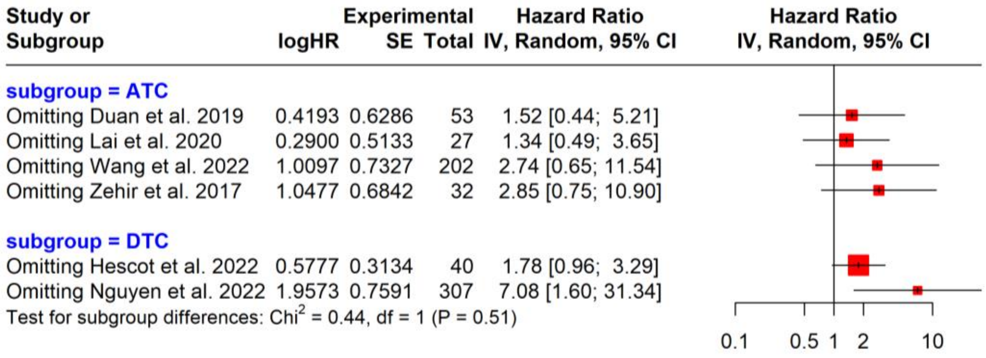

Figure S4: Sensitivity analysis for TP53 on OS

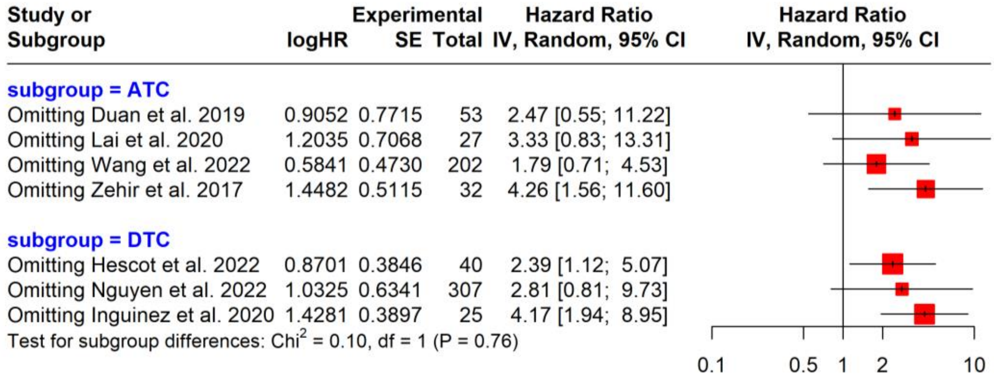

Supplement: Supplementary file 1 [file cancers-17-00939-s001.zip › Supplement S2_Sensitivity analysis.pdf]
